# Serum adipokines as non-invasive biomarkers in Crohn’s disease

**DOI:** 10.1038/s41598-020-74999-6

**Published:** 2020-10-22

**Authors:** Lorena Ortega Moreno, Ancor Sanz-Garcia, Marina J. Fernández de la Fuente, Ricardo Arroyo Solera, Samuel Fernández-Tomé, Alicia C. Marin, Irene Mora-Gutierrez, Paloma Fernández, Montserrat Baldan-Martin, María Chaparro, Javier P. Gisbert, David Bernardo

**Affiliations:** 1 Departamento de Medicina, Universidad Autónoma de Madrid, Madrid, Spain; 2 Servicio de Aparato Digestivo, Hospital Universitario de la Princesa, Instituto de Investigación Sanitaria Princesa (IIS-IP), Madrid, Spain; 3 Centro de Investigación Biomédica en Red de Enfermedades Hepáticas y Digestivas (CIBERehd), Madrid, Spain; 4 Unidad de Análisis de Datos, Hospital Universitario de la Princesa, Instituto de Investigación Sanitaria Princesa (IIS-IP), Madrid, Spain; 5 Universidad de Alcalá, Alcalá de Henares, Madrid, Spain; 6 Instituto de Medicina Molecular Aplicada (IMMA), Facultad de Medicina, Universidad San Pablo CEU, Montepríncipe, Madrid, Spain; 7 Mucosal Immunology Laboratory, Instituto de Biología y Genética Molecular (IBGM), Universidad de Valladolid-CSIC, c/ Sanz y Forés 3, 47003 Valladolid, Spain

**Keywords:** Molecular biology, Biomarkers, Gastroenterology

## Abstract

Adipose tissue secretes molecules that can promote activity in Crohn’s disease. We aimed to evaluate the role of serum adipokines as possible biomarkers in Crohn’s disease. Serum samples were obtained from 40 patients with endoscopically active or quiescent Crohn’s disease and 36 healthy controls. Serum leptin, ghrelin, resistin and adiponectin levels were analysed by Multiplex in a Luminex 200 system technology. Receiver Operating Characteristic curves were performed to evaluate the adipokines discriminatory capacity. A logistic regression adjusted by possible confounders (i.e. gender, age, BMI) was performed for those adipokines that showed an area under the curve > 0.7. No differences were found in age, gender or BMI among groups. Distribution for serum resistin was different among the three groups of study, and only this adipokine showed an area under the curve of 0.75 comparing actives patients and healthy control groups. Resistin median concentration was selected as a cut-off for a logistic regression analysis; odds ratio along its 95% confidence interval adjusted by gender, age, and BMI yielded a value of 5.46 (1.34–22.14) comparing actives patients and healthy controls. High concentration of serum resistin is probably associated to activity, being this association independent of gender, age or BMI.

## Introduction

Inflammatory bowel disease (IBD) is a pathology associated to Occidentalized countries. However, its incidence is currently increasing over the world and it may become a global disease^1^. Hence, the cost to the Public Health System has been incremented due to its chronicity and the early aged-onset of this pathology^2–4^. IBD is classified in two different entities named Crohn’s disease (CD) and ulcerative colitis that are characterized by a chronic inflammation of the gastrointestinal tract. While ulcerative colitis is only limited to the colon, CD may come along with extra intestinal manifestations^5^. Under this scenario, it is necessary to find new strategies for the improvement of IBD patients’ quality of life.

Visceral adipose tissue is a risk factor for pathologies like diabetes *mellitus* type 2 and cardiovascular disease^6^, but also for CD where it increases its grade of inflammation^7^. Indeed, there is a mesenteric adipose tissue enlargement in CD, known as *creeping fat*^8^ where inflamed intestinal zones show increased numbers of small size adipocytes and immune cells^9^. This *creeping fat* can modulate the immune system inducing an inflammatory response by secretion of several adipokines including leptin, adiponectin or resistin^10^.

Leptin is an adipokine secreted by adipocytes which stimulates the production of pro-inflammatory IL-1β and IL-6 in T cells^11^. Adiponectin is secreted from adipocytes and displays anti-inflammatory properties^12^. On the other hand, resistin is an adipokine upregulated in the mesenteric adipose tissue from CDpatients^11^ which may act as an independent predictor of disease in CD^13^. Ghrelin is an endogenous ligand of the growth hormone secretagogue receptor^14^ and anorexigenic peptide with anti-inflammatory activity^15^ reason why it may also be marker of disease activity in CD^16^.

Despite the role that adipokines and the lipid metabolism elicit in CD pathogenesis, to our knowledge few studies explored them as non-invasive biomarkers to assess mucosal status in patients with endoscopically inflamed or active CD (aCD) and non-inflamed or quiescent disease (qCD). Our aim therefore was to analyse the adipokine serum profile in CD patients and healthy controls (HC) in order to get a deeper insight into their contribution towards CD pathogenesis and to assess CD mucosal status (quiescent or active)in the absence of a colonoscopy.

## Results

### Descriptional study

**Figure 1:**
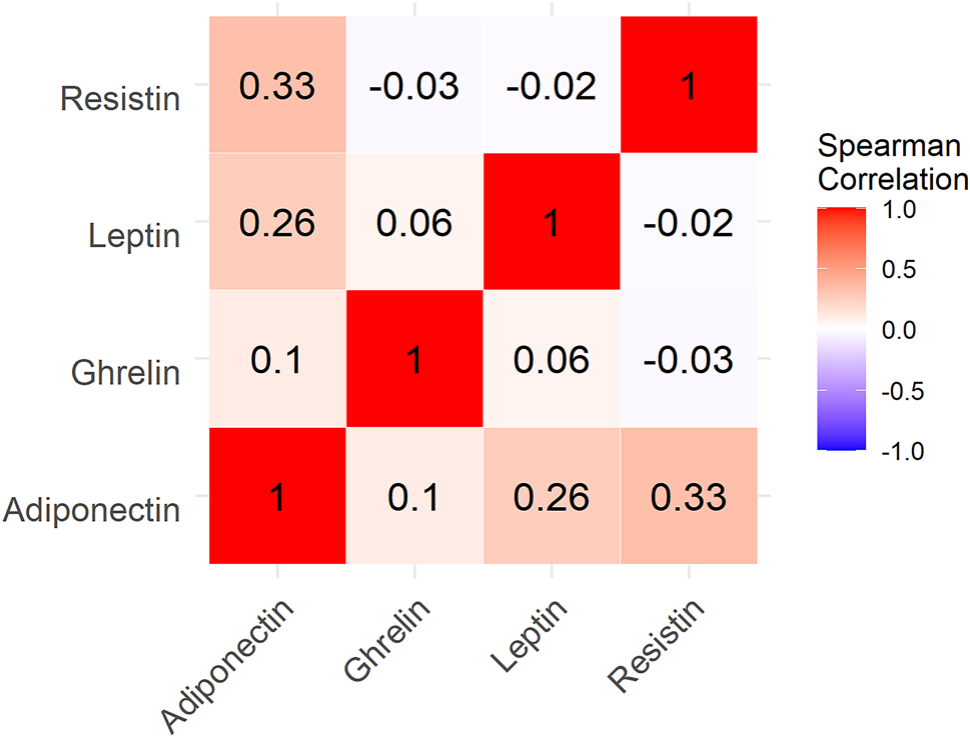
Spearman correlations for adipokines.

There were no differences in gender, age and BMI among study groups (Table 1). Following exclusion of the samples with levels above or under detection limits, a total of 2 HC were removed. Results from the 74 remaining individuals are shown in Table 2. Median comparisons of the adipokines concentrations (natural log transformed) among groups were only statistically significant in the case of resistin (p = 0.04) (Table 2), while Dunn test post hoc analysis revealed that this was due to the comparison between aCD and HC (p = 0.03). No correlation was found among the four serum adipokines levels (Fig. 1).

**Table 1:** Baseline characteristics in the study cohort (n = 76).

Variable	HC (36)	qCD (22)	aCD (18)	p
Age(years)	51.4 ± 15.1	46.5 ± 12.2	44.4 ± 13.2	0.2
BMI	24.4 ± 3.9	24.1 ± 4.6	26.4 ± 3.4	0.1
Males(n, %)	16, 44.4	11, 50	8, 44.4	0.9

Continuous variables were reported as mean ± SD.

Chi square test and means comparisons were performed.

*HC* healthy control, *qCD* quiescent CD, *aCD* active CD, *BMI* body mass index.

**Table 2:** Adipokines concentration in the groups of the study and Kruskal–Wallis comparison (n = 74).

Molecule(pg/ml)	HC (34)	qCD (22)	aCD (18)	p
Adiponectin	17.7 (17.1–17.8)	17.7 (17.6–17.8)	17.7 (17.6–17.8)	0.3
Resistin	9.0 (8.8–9.3)	9.2 (8.9–9.5)	9.3 (9.2–9.7)	0.04
Leptin	7.8 (6.8–9.0)	7.5 (6.4–8.7)	7.9 (6.7–8.7)	0.9
Ghrelin	4.6 (4.0–6.3)	4.8 (3.9–5.5)	3.9 (3.6–5.4)	0.07

Variables were reported as median (interquartile range); Kruskal–Wallis comparisons were performed. In the case of resistin, Dunn test post hoc analysis was significant only for HC vs. aCD(p = 0.03). Only those patients with data for the four adipokines variables were considered. All variables were natural log transformed before the analysis.

*HC* healthy control, *qCD* quiescent CD, *aCD* active CD.

### Discriminatory power of adipokines

**Figure 2:**
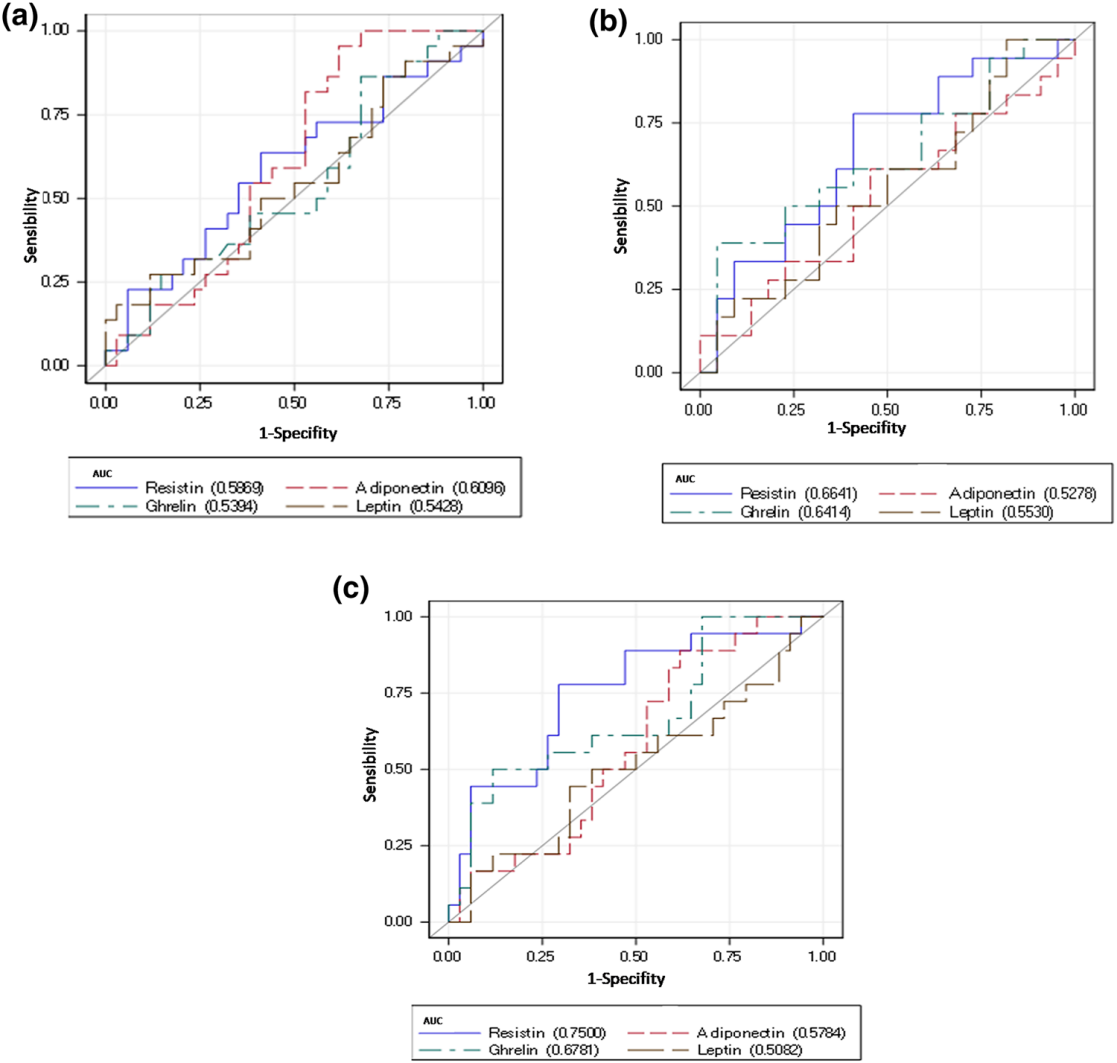
**(a)** Receiving operating characteristic curves for leptin, ghrelin, adiponectin and resistin comparing patients with quiescent Crohn’s disease (qCD) and healthy controls (HC). (**b)** Receiving operating characteristic curves for leptin, ghrelin, adiponectin and resistin comparing patients with quiescent Crohn’s disease (qCD) and active Crohn’s disease (aCD). (**c)** Receiving operating characteristic curves for leptin, ghrelin, adiponectin and resistin comparing patients with active Crohn’s disease (aCD) and healthy controls (HC).

**Figure 3:**
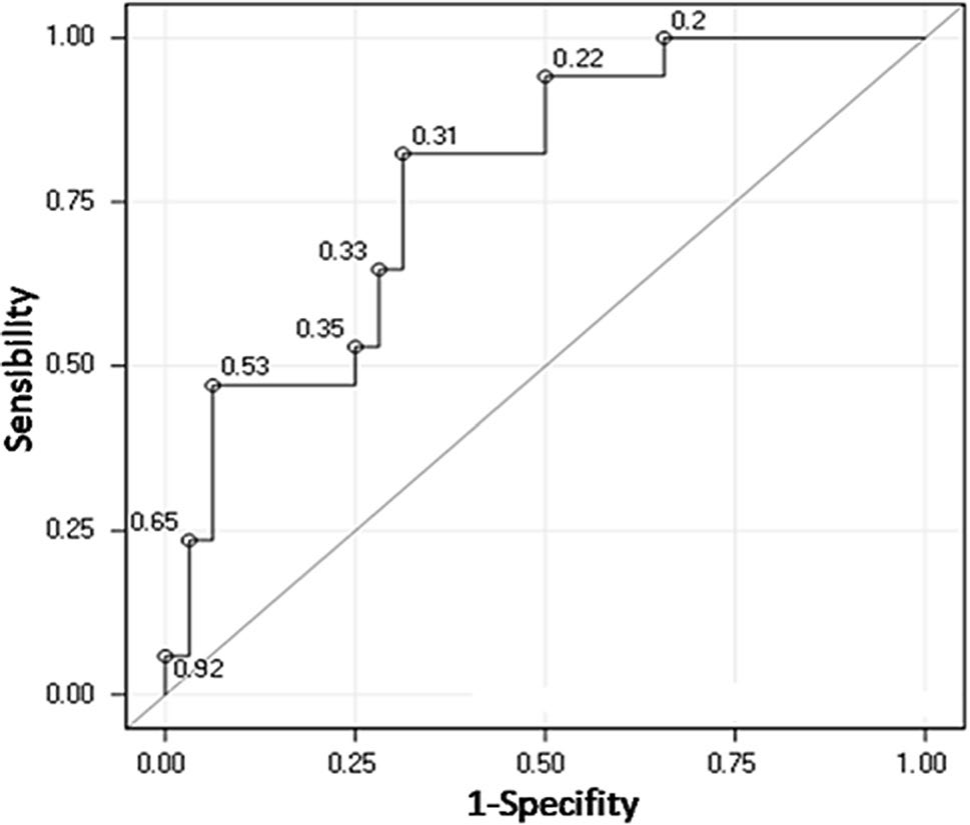
Youden indexfor resistin in the receiving operating curve comparing patients with active Crohn’s disease (aCD) and healthy controls (HC).

The discriminatory power of leptin, adiponectin, ghrelin and resistin between the study groups was tested by ROC curves. AUC was below of 0.7 for comparisons between qCD and HC (Fig. 2a, Table 3a), but also between aCD and qCD (Fig. 2b, Table 3b). The discriminatory power of each adipokine between aCD and HC, was also tested by ROC curves analysis, and the AUC was above 0.7 only for resistin (Fig. 2c), AUC along with its 95% confidence interval was 0.75 (0.61–0.89), comparing aCD and HC (Table 3c). Following with this analysis, Youden cut-off index for ROC curve (Fig. 3) matched with the total resistin median concentration (9822 pg/ml), so this value worked as a cut-off for subsequent regression analysis between resistin and CD activity, which showed a strong association, odds ratio (OR) along with its 95% confidence interval was 5.46 (1.34–22.14) adjusted by gender, age and BMI; thus, patients with a resistin concentration above 9822 pg/ml may be candidates for developing activity.

**Table 3:** Receiving operating characteristic statistics.

Adipokine (pg/ml)	ROC statistics	
	AUC	95% CI
**(a) From the comparison between qCD and HC**		
Resistin	0.59	0.43–0.74
Adiponectin	0.61	0.46–0.76
Ghrelin	0.54	0.38–0.69
Leptin	0.54	0.38–0.70
**(b) From the comparison between qCD and aCD**		
Resistin	0.66	0.49–0.83
Adiponectin	0.53	0.34–0.71
Ghrelin	0.64	0.46–0.82
Leptin	0.55	0.37–0.74
**(c) From the comparison between aCD and HC**		
Resistin	0.75	0.61–0.89
Adiponectin	0.58	0.42–0.74
Ghrelin	0.68	0.52–0.84
Leptin	0.51	0.33–0.68

*AUC* area under the curve along with their 95% confidence interval (CI), *HC* healthy control, *aCD* active CD.

## Discussion

*Creeping fat* and mesenteric adipose tissue are characteristic of CD^17^. Indeed, this adipose tissue has the capacity to produce several adipokines which participate in the intestinal inflammatory response^18^. In this regard, and in order to get a deeper insight into their contribution towards IBD pathogenesis, we decided to study their serum levels in CD patients, both endoscopically active and quiescent. Hence, our results describe how high resistin serum levels are associated with endoscopically active (inflamed) CD patients.

So far, studies of association among adipokines and IBD are contrasting. Leptin has been described asincreased^19^, reduced^20^ and even unaltered^21^ in IBD patients. In this regard, and although leptin acts as a proinflammatory adipokine, results in clinical studies about its role in IBD pathogenesis are ambiguous and, in agreement with our findings, several studies found that it was not related to IBD^21,22^.

Results regarding adiponectin involvement in IBD pathogenesis are also divergent^12,23^. This adipokine is characterized by anti-inflammatory properties and its concentration decreases in obesity^24^. However, adiponectin has been associated to cardiovascular mortality in patients with type 2 diabetes playing a paradoxical role as the higher concentration of adiponectin the higher is the risk of cardiovascular mortality^25,26^. Here, there was no association with activity in CD and further studies are necessary to test its role in CD.

Ghrelin has been introduced as an orexigenic peptide, being our results in the line of those from Nishi et al*.* who did not show any change in serum ghrelin in CD patients^21^.

Resistin, on the contrary, is an adipokine that participates in several inflammatory processes. Indeed, it has been associated with impairment of type 2 diabetes and atherosclerosis^27^. In our study, resistin showed an association with disease activity in CD regardless of the small size of the data cohort. Hence, our results suggest that this adipokine could play an important role in IBD pathogenesis at the time that its serum levels could have a role as a non-invasive biomarker to assess mucosal status of CD patients in the absence of a colonoscopy.

Obesity has been associated with IBD prevalence being more common in CD than in ulcerative colitis. However, how obesity affects the development of the pathology has not been elucidated yet^28^. The Nurses’ Health Study, from USA, revealed that obesity assessed by BMI was associated with a higher risk of CD than ulcerative colitis^29^. In our study, there are no significant differences among groups when BMI was compared.

We are aware about the limitations of our study as given the restricted sample size of our cohort; we could not find important differences due to the lack of power. Another limitation is the absence of measures regarding visceral adipose tissue, reason why we used BMI as a measure of total fat. Our results reinforce the role of resistin as a possible biomarker of disease activity in CD and a novel target to elucidate CD pathogenesis.

Further studies should complement our approach including larger and independent cohorts, as well the measure of visceral adipose tissue in order to investigate how resistin, and other adipokines, might lead to an inflamed state in CD and to unravel new functions of serum adipokines which could work as serum biomarkers of endoscopic activity in CD.

## Conclusion

We analyze here four adipokines that have been associated with inflammation and chronic diseases along the literature. Our aim was to elucidate if they are able to work as biomarkers of activity in CD. Among them, only high serum resistin levels seems to be associated with activity in CD. Resistin would be a candidate for future studies of association with activity regarding this type of IBD. It is necessary to analyze this molecule in an independent and larger cohort in the future to test its capacity as biomarker of inflamed state in CD.

## Methods

A total of 76 human serum samples were analyzed. The biological samples used in this project were obtained from the Collection of Biological Samples of Dr. Javier P. Gisbert, which is registered in the National Biobanks Registry of Instituto de Salud Carlos III (C.0003482), Madrid, Spain. The study protocols and the informed consent procedures were approved by the Institutional Ethic Committee of Hospital Universitario de La Princesa, Madrid, Spain. All participants in the study gave written informed consent. All methods were carried out in accordance with the approved guidelines.

All serum samples were obtained from CD patients and HC at the moment of colonoscopy. The Simple Endoscopic Score for Crohn’s Disease (SES-CD) was determined during colonoscopy in all CD patients in order to classify them as aCD (SES-CD ≥ 3) or qCD (SES-CD ≤ 2). All colonoscopies were performed in the context of the normal clinical practice for CD diagnose or monitoring. Inclusion criteria for patients with CD were patients older than 18 years old diagnosed with CD according to European Crohn’s and Colitis Organisation criteria and did not meet one or more exclusion criteria. Exclusion criteria for patients with CD were the following: to have received immunosuppressive treatment for any pathology other than IBD; to have an immune-mediated disease, different from IBD; to have a neoplasm or an active infection at the time of the colonoscopy; to be pregnancy or lactation at the moment of the colonoscopy.

HC were referred to colonoscopy due to changes in the bowel transit, rectal bleeding or colorectal cancer screening. However, in all cases they had normal (non-inflamed) intestinal mucosa and had not known inflammatory processes, autoimmune diseases or malignancies, not pregnancy nor lactation and without any infectious diseases at the moment of the colonoscopy.

Serum samples were obtained following blood centrifugation, aliquoted and immediately cryopreserved at − 80 °C. Samples had not been defrosted before the experiment to make sure that all of them were analysed in the same conditions. Samples from a total of 36 HC, 18 patients with aCD and 22 patients with qCD were analysed. Patients’ information regarding gender, age, ethnic background (all of them were caucasians) and BMI were collected in a codified manner from medical records. Demographic variables (i.e. gender, age and BMI) were analysed by chi square test (frequencies) or ANOVA test (means).

Adipokines from serum samples were analysed by duplicated using Bio-PlexPro Human Diabetes group (leptin, ghrelin and resistin) and Bio-plexPro Hu Diab Adiponectin (adiponectin) from Bio-Rad (Hércules, CA, USA), in a Luminex200 System technology. Samples were processed following manufacturer’s instructions. The final concentration value of each adipokine was the result of the mean from the two duplicated measures. Adipokines concentrations above or below the kit sensitivity range were not considered. Skewness was tested by Kolgomorov–Smirnov test. Adipokines levels were all natural log transformed. Spearman correlation was performed for the adipokines. Median and interquartile range for each patient group was determined for the adipokines and Kruskal–Wallis test was performed. Receiver Operating Characteristic (ROC) curves and the area under the curve (AUC) were carried out to evaluate the discriminatory capacity of the adipokines levels comparing different study groups. The Youden cut-off index got from the ROC curve was analysed. Furthermore, those adipokines that showed an AUC > 0.7 along their 95% confidence interval were selected for a binary logistic regression adjusted by possible confounders (i.e. gender, age, BMI) in order to test their possible association with CD. All statistical analyses were carried out in SPSS v15, SAS University Edition and R.

## Data Availability

Datasets are available from the corresponding author on reasonable request.

## Abbreviations

aCD: Active Crohn’s disease
qCD: Quiescent Crohn’s disease
CD: Crohn’s disease
IBD: Inflammatory bowel disease
HC: Healthy control
SES-CD: Simple Endoscopic Score for Crohn’s Disease
ROC: Receiver operating characteristic
AUC: Area under the curve
